# Effect of the *Melhor em Casa* program on hospital costs

**DOI:** 10.11606/s1518-8787.2019053000859

**Published:** 2019-11-18

**Authors:** Fábio Nishimura, Aniela Fagundes Carrara, Carlos Eduardo de Freitas

**Affiliations:** I Universidade Federal de Mato Grosso. Curso de Ciências Econômicas. Programa de Pós-Graduação em Economia. Rondonópolis, MT, Brasil

**Keywords:** Home Care Services, Economics, Health Care Costs, Program Evaluation, Unified Health System

## Abstract

**OBJECTIVE:**

To verify if the *Melhor em Casa* program can actually reduce hospitalization costs.

**METHODS:**

We use as an empirical strategy a Regression Discontinuity Design, which reduces endogeneity problems of our model. We also performed tests of heterogeneous responses and robustness. Data on the dependent variable, namely hospitalization costs, were collected in the Department of Informatics of the Unified Health System (DATASUS), using the microdata set from the Hospital Admissions System of the Unified Health System (SUS) from 2010 to 2013, totaling 3,609,384 observations. The covariates or control variables used were age and costs with patients in the intensive care unit, also from DATASUS.

**RESULTS:**

The results point out that the *Melhor em Casa* program effectively reduced hospitalization costs by approximately 4.7% in 2011, 5.8% in 2012 and 10.2% in 2013.

**CONCLUSIONS:**

Based on the analyses, we observed that maintaining the program can effectively improve the management of public resources, since it reduced the hospitalization costs in the three years studied. The program reduced hospitalization costs of risk groups and also in situations that usually increase hospital costs such as lack of equipment and elective hospitalizations. Thus, it can be affirmed that the program can reduce hospitalization costs, especially in risk and more vulnerable groups, showing efficiency as a public policy.

## INTRODUCTION

Our study verified if the *Melhor em Casa* program (MemC) can actually reduce hospitalization costs. Hospital care is understood as an important form of social policy for life maintenance and a mandatory form of restoring constitutional principles to the Brazilian population. Hospital care should be effectively provided to avoid a system collapse, especially with a limited budget.

In 2011, the federal government created the MemC to control hospitalization problems and reduce unnecessary costs for the population^1 , 2^ . The program aims to articulate with home care (HC) – that is, the patients admitted to the health unit and ready to complete their recovery in the comfort of their household, with the relatives, can be transferred. Thus, hospitalization costs reduce and hospital mortality decreases.

However, we could not see the commitment of health managers. We observed cases of maladministration and poor technical and structural conditions instead, which reduce the efficiency of hospital services. The consequences are unrecoverable economic and life-related costs^3^ .

One of the major problems of hospital care is the cost of services provided in the health unit, with high waste, which reaches R$ 3.6 billion a year with consultations, hospitalizations and unnecessary exams. The complex composition of hospital costs result in waste and inefficiency in the public service^9^ . In addition to verify the effect of MemC, our article also contributes to the application of the empirical strategy of the regression discontinuity design (RD), which guarantees responses without endogenous bias, confirming or not the program efficiency.

The tests of heterogeneous responses and robustness were used to verify if the estimators were accurate. The main hypothesis of our study was that the program can reduce hospitalization costs, making public hospital management more efficient.

## METHODS

Data on the dependent variable, namely hospitalization costs, were collected in the Department of Informatics of the Unified Health System (DATASUS), using the microdata set of the Hospital Admission system of the Brazilian Unified Health System (SUS) from 2010 to 2013, totaling 3,609,384 observations in the three years of analysis. The values of the variable of interest, hospitalization costs, were deflated; thus, the series suffers real impact without inflationary influence.

In 2011, the average hospitalization cost was R$ 38.67 per patient/day in the municipalities covered by the program. In 2013, this value reached R$ 42.78 per patient/day, a 10.6% increase within three years. In municipalities not covered, the average was R$ 30.83 per patient/day in 2011 and R$ 33.51 in 2013, an 8.7% increase within three years. Based on these data, we can affirm this program is indicated to municipalities with higher hospitalization costs.

Data were collected in Datasus and the National Register of Health Establishments (NRHOSP), both organs of the Ministry of Health, to analyze the effects of MemC. We used a dummy to identify the municipalities covered by the MemC. They were named “treated” and coded with the value 1, while the municipalities not covered were named “controls” and coded with the value 0.

Based on the data, we observed the program included 23 of the 5,570 Brazilian municipalities in 2011, increasing to 90 municipalities in 2012, and to 184 in 2013. The coverage rate increased from 0.41% of the Brazilian territory in 2011 to 3.3% in 2013.

The covariates or control variables used were age and hospitalization costs of patients in the intensive care unit. These data were obtained from Datasus database, from 2010 to 2013. The control variables are used to greater precision to the estimated values^10^ , but do not interfere or generate any bias in the results.

Our article analyzed how home care (in this case, MemC program) affects the hospitalization costs. As an empirical strategy, we used the regression discontinuity design^11^ due to the adherence criterion related to a cutoff point and exogenous probability, namely “cutoff.” The established cutoff were municipalities with more than 20,000 inhabitants. Thus, we estimate the following equation:

$$Y_{imp}\;=\;\beta_{0}\;+\;\beta_{1}\;MemC_{imp}\;+\;\beta_{2}\;T_{imp}\;+\;\varepsilon_{imp}$$ (1)

In which *Y*_*imp*_ is the variable of interest of the model for the individual *i* , in the municipality *m,* and in the year *p* ; MemC is the *Melhor em Casa* program, which takes value equal to 1, if the individual’s municipality is covered by the program, and 0 if it is not covered, for the individual *i* , in the municipality *m,* and the year *p* ; The *T*_*ip*_ indicates if the municipality is above or below the cutoff, cited previously, for the individual *i* , in the municipality *m,* and in the year *p* ; Finally, *ε*_*ip*_ is an error term. Due to the sensitivity of considering an increase in probability – but not from zero to one, since the attribution to treatment may depend on additional factors –, the regression discontinuity model fuzzy was applied *.* This ensures a causal identification among the selected variables, enabling the confirmation of the effect of MemC on hospitalization costs with higher statistical security.

To perform local regressions, you need to enter a window (bandwidth) that will determine the size of the cutoff distance at the level of sample observations. Then, we used the methodology of Calonico et al.^16^ This variation of bandwidth is also a way to guarantee our empirical strategy, emphasizing that we test our model for a linear and quadratic specification.

In addition to the aforementioned tests, we applied three more robustness tests to confirm our empirical strategy. First, we verified possible effects of other programs or actions of years prior to the beginning of the program. Another test was the cutoff alteration *,* that is, we made our regressions with different cutoffs than the one stipulated by the program. Finally, we tested the covariates of our model. The tests should not generate statistically significant estimators, guaranteeing the parameters of our model.

We also performed heterogeneous test responses to understand and confirm the effects of MemC on sub-sample situations, namely risk groups (pregnant and older adults), which require a longer hospitalization time. We analyzed if the program can affect municipalities with fewer working equipment than the national average, which would cause a longer hospitalization due to the delay in the results of the exams. Finally, we verified the effect of MemC on the group of people hospitalized without urgency, or elective, which would show the speed of care and the reduction of costs with this type of hospitalization.

## RESULTS

We initially verified if discontinuity occurs at the 20,000 inhabitants’ cutoff point to guarantee the statistical results. The Figure shows the result of the initial test, in which a discontinuity is observed: the amount spent on hospital admissions reduced. It is an important criterion, since this criterion ensures our results come from a causal relationship and from the comparison that we can perform between municipalities close to the cutoff point (point 0 of the Figure ) and that have as a difference only the coverage or not of the program.

**Figure:**
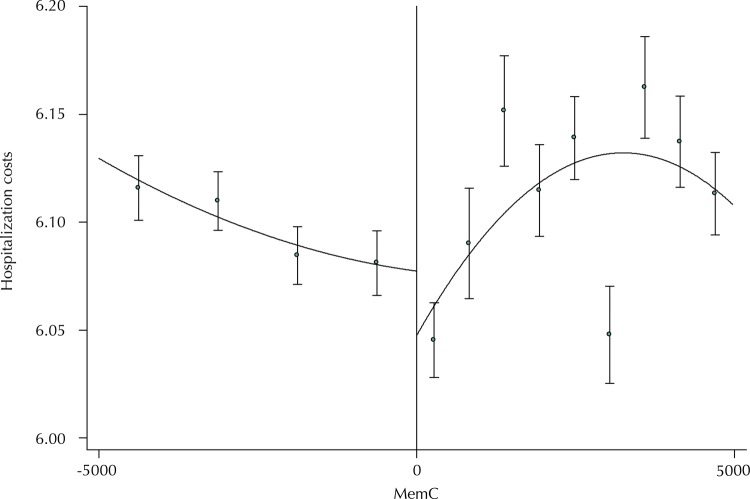
The discontinuity of the program in the cutoff

Table 1 shows the discontinuity, which was a basic factor to calculate our estimators, with the results of all regressions indicating negative and statistically significant estimators. The reductions in the hospitalization costs reached 4.7% (p < 0.01). This effect remained in the subsequent years, reducing up to 10.2% (p < 0.01).

**Table 1:** Effect of *Melhor em Casa* program on hospitalization costs, 2010 to 2013.

Variable	1	2	3	4	5	6
*MemC*_t_	-0.044^h^	-0.047^h^	-0.007	-0.035^j^	-0.034^j^	-0.007
	(0.016)	(0.016)	(0.005)	(0.020)	(0.020)	(0.007)
*MemC*_t+1_	-0.048^i^	-0.058^h^	-	-0.044^i^	-0.055^i^	-
	(0.019)	(0.021)		(0.023)	(0.024)	
*MemC*_t+2_	-0.101^h^	-0.102^h^	-	-0.098^h^	-0.096^h^	-
	(0.018)	(0.019)		(0.019)	(0.020)	
Specification	Linear	Linear	Linear	Quad	Quad	Quad
Bandwidth	msetwo	msecomb2	msetwo	msetwo	msecomb2	msetwo
Controls	Yes	Yes	Yes	Yes	Yes	Yes

No. Obs	182.350	182.350	182.350	182.350	182.350	182.350

^a^ All specifications use triangular Kernel.

^b^ All specifications use triangular Kernel.

^c^ MemC ( *Melhor em casa* ) estimates the discontinuity of municipalities immediately above 20,000 inhabitants.

^d^ Msetwo: two different MSE-optima L Bandwidth selectors and MSECOMB2: two different MSE-optima L Bandwidth selectors for median, in which they refer to selectors-bandwidth Optimum Calonico et al.^16^

^e^ Columns 1, 2, 4, and 5 represent the main estimates of our specified model. Columns 3 and 6 refer to statistical hypothesis test of a year prior to the start of MemC.

^f^ Robust standard errors in parentheses.

^g^ Statistical significance level represented by: (h) p < 0.01, (i) p < 0.05 and (j) p < 0.10.

^l^ Quad: quadratic function

Regarding the heterogeneous responses, based on the estimators shown in Table 2 , Panel A, we concluded that the program reduced the hospitalization costs of women with risk pregnancies within three years after its beginning, resulting in a cost saving up to 9.3% (p < 0.01). In panel B, regarding the old adults, hospitalization costs reduced up to 9.6% (p < 0.01) within the three years. Thus, the program shows that the reduction in costs reaches risk and more vulnerable groups.

**Table 2:** Effect of *Melhor em Casa* program on hospitalization costs, 2011 to 2013.

Variable	1	2	3	4
**Panel A:** Effect on pregnant women at risk				
*MemC*_t_	-0.037^h^	-0.040^g^	-0.041^g^	-0.042^g^
	(0.016)	(0.016)	(0.017)	(0.017)
*MemC*_t+1_	-0.048^h^	-0.059^g^	-0.045^h^	-0.055^h^
	(0.021)	(0.021)	(0.023)	(0.024)
*MemC*_t+2_	-0.092^g^	-0.090^g^	-0.093^g^	-0.092^g^
	(0.018)	(0.019)	(0.019)	(0.020)

No. Obs.	166,295	166,295	166,295	166,295

**Panel B:** Effect on older adults				
*MemC*_t_	-0.067^h^	-0.067^g^	-0.073^h^	-0.074^h^
	(0.031)	(0.015)	(0.037)	(0.037)
*MemC*_t+1_	-0.073^h^	-0.064^i^	-0.085^g^	-0.062^i^
	(0.033)	(0.036)	(0.034)	(0.036)
*MemC*_t+2_	-0.096^g^	-0.111^g^	-0.086^h^	-0.079^i^
	(0.035)	(0.040)	(0.040)	(0.043)
Specification	Linear	Linear	Quad	Quad
Bandwidth	msetwo	msecomb2	msetwo	msecomb2
Controls	Yes	Yes	Yes	Yes

N. Obs.	166,295	166,295	166,295	166,295

^a^ All specifications use triangular Kernel.

^b^ MemC ( *Melhor em Casa* ) estimates the discontinuity of municipalities immediately above 20,000 inhabitants.

^d^ Msetwo: two different MSE-optima L Bandwidth selectors and MSECOMB2: two different MSE-optima L Bandwidth selectors for median, in which they refer to selectors-bandwidth Optimum Calonico et al.^16^

^d^ Columns 1.2, 3 and 4 represent estimations as heterogeneous responses.

^e^ Robust standard errors in parentheses.

^f^ Statistical significance level represented by: (g): p < 0.01, (h): p < 0.05 and (i): p < 0.10.

^l^ Quad: quadratic function

Table 3 , Panel A, shows that in the first three years the reduction in hospitalization costs ranged between 4.4% and 11.7%. Both reductions were statistically significant at 1%, even considering the municipality with a fewer equipment than the national average – which hinders the patient’s release, as it also delays the delivery of the results of the exams, resulting in higher costs. Table 3 , Panel B, shows a reduction between 6.7% (p < 0.1) and 17.9% (p < 0.01), indicating an economy in elective hospitalizations.

**Table 3:** Effect of the *Melhor em Casa* program on hospitalization costs – municipalities with fewer hospital equipment and elective hospitalizations, 2011 to 2013.

Variable	1	2	3	4
**Panel A:** Effect on municipalities with fewer hospital equipment				
*MemC*_t_	-0.049^g^	-0.055^g^	-0.046^h^	0,044^h^
	(0.016)	(0.016)	(0.020)	(0.020)
*MemC*_t+1_	-0.048^h^	-0.057^g^	-0.044^i^	-0.055^h^
	(0.021)	(0.021)	(0.023)	(0.024)
*MemC*_t+2_	-0.110^g^	0,117^g^	-0.106^g^	-0.101^g^
	(0.018)	(0.020)	(0.019)	(0.019)

No. Obs.	182.350	182.350	182.350	182.350

**Panel B:** Effect on elective hospitalizations				
*MemC*_t_	-0.085^g^	-0.071^h^	-0.161^g^	-0.134^g^
	(0.032)	(0.030)	(0.041)	(0.041)
*MemC*_t+1_	-0.067^i^	-0.068^i^	-0.075^i^	-0.085^i^
	(0.038)	(0.038)	(0.040)	(0.044)
*MemC*_t+2_	-0.125^g^	-0.122^g^	-0,171^g^	-0.179^g^
	(0.042)	(0.046)	(0.047)	(0.047)
Specification	Linear	Linear	Quad	Quad
Bandwidth	msetwo	msecomb2	msetwo	msecomb2
Controls	Yes	Yes	Yes	Yes

N. Obs.	46,916	46,916	46,916	46,916

^a^ All specifications use triangular Kernel.

^b^ MemC ( *Melhor em Casa* ) estimates the discontinuity of municipalities immediately above 20,000 inhabitants.

^d^ Msetwo: two different MSE-optima L Bandwidth selectors and MSECOMB2: two different MSE-optima L Bandwidth selectors for median, in which they refer to selectors-bandwidth Optimum Calonico et al.^16^

^d^ Columns 1.2, 3 and 4 represent estimations as heterogeneous responses.

^e^ Robust standard errors in parentheses.

^f^ Statistical significance level represented by: (g): p < 0.01, (h): p < 0.05 and (i): p < 0.10.

^l^ Quad: quadratic function

In addition to the heterogeneous responses, we applied robustness tests to statistically guarantee the assertion of the initial hypothesis. In Table 1 , columns 3 and 6, we tested the possible occurrence of effects in years prior to the beginning of the program. We observed no statistical significance, without evidence of any effect of any external factor and before the beginning of the MemC in hospitalization costs. This reinforces that the program is the main factor for cost reduction.

We continue with the robustness tests in Table 4 . Panels A and B show that the results are not statistically significant when creating false cutoffs of 10,000 and 50,000 inhabitants. This was expected, since it certifies that the effects occur only in the cutoff criterion determined by the program, which is 20,000 inhabitants, showing that it did not occur by a statistical accident.

**Table 4:** Robustness tests of the specified model, 2011 to 2013.

Variable	1	2	3	4
**Panel A:** Cutoff alteration for 10,000 inhabitants				
*MemC*_t_	-0.020	-0.029	-0.005	-0.031
	(0.015)	(0.020)	(0.017)	(0.021)
*MemC*_t+1_	0.009	-0.016	0.033	-0.006
	(0.041)	(0.055)	(0.041)	(0.068)
*MemC*_t+2_	0.026	0.052	0.046	−0.048
	(0.035)	(0.037)	(0.039)	(0.053)
No. Obs.	215,393	215,393	215,393	215,393
**Panel B:** Cutoff alteration for 50,000 inhabitants				
*MemC*_t_	0.012	0.002	0.033	0.028
	(0.020)	(0.017)	(0.021)	(0.019)
*MemC*_t+1_	-0.0002	-0.018	0.038	0.005
	(0.033)	(0.061)	(0.035)	(0.094)
*MemC*_t+2_	0.029	0.025	0.041	0.033
	(0.022)	(0.022)	(0.026)	(0.024)

No. Obs.	400,834	400,834	400,834	400,834

**Panel C:** Test on Covariates				
Age	-0.013	0.357	-0.051	3.482
	(1.842)	(1.698)	(2.423)	(2.460)
Days of hospitalization in the ICU	0.090	0.092	0.096	0.102
	(0.056)	(0.054)	(0.075)	(0.072)
Specification	Linear	Linear	Quad	Quad
Bandwidth	msetwo	msecomb2	msetwo	msecomb2
Controls	Yes	Yes	Yes	Yes

No. Obs.	182.350	182.350	182.350	182.350

^a^ All specifications use triangular Kernel.

^b^ MemC ( *Melhor em Casa)* estimates the discontinuity of municipalities with more than 20,000 inhabitants, for the cases of Panel C.

^d^ Msetwo: two different MSE-optima L Bandwidth selectors and MSECOMB2: two different MSE-optima L Bandwidth selectors for median, in which they refer to selectors-bandwidth Optimum Calonico et al.^16^

^d^ Columns 1, 2, 3, and 4 represent estimates as robustness tests.

^e^ Robust standard errors in parentheses.

^f^ Statistical significance level represented by: (g): p < 0.01, (h): p < 0.05 and (i): p < 0.10.

^l^ Quad: quadratic function

Concluding our tests, in Table 4 , Panel C, we tested the control variables, but we did not have statistical significance in any estimator, which establishes that MemC is the only factor to affect the reduction in hospitalization costs. Thus, after verifying all the results, we can conclude that the program reduces hospitalization costs.

## DISCUSSION

Our study analyzed how the *Melhor em Casa* program affected the hospitalization costs. The program proposes the provision of services by teams composed of several professionals in the health area to promote patient recovery in the comfort of their household (home care). Besides the recovery in a healthier environment, it also promotes hospital bed vacancy, reducing daily costs of hospitalization.

After ensuring that the results present causal characteristics, we verified that the MemC can effectively reduce hospitalization costs, with reductions in approximately 4.7% in 2011, 5.8% in 2012 and 10.2% in 2013. The results heterogeneous responses and the robustness test were the ones expected, confirming the results of our model and statistically supporting our estimators.

We could observe that the risk groups (pregnant women and older adults), even with higher costs due to specific care, also had their costs reduced with the program. MemC can effectively reduce hospitalization costs of municipalities with fewer hospital equipment than the national average. The program can also be efficient when considered the elective hospitalizations, that is, those that are not urgent. Thus, we conclude that the program helps reduce hospitalization costs, especially in risk and more vulnerable groups, showing to be an efficient public policy.

## References

[1] 1. Ministério da Saúde (BR). Manual instrutivo do melhor em casa. Brasília, DF; 2011 [citado 1 mai 2017]. Disponível em: http://189.28.128.100/dab/docs/geral/cartilha_melhor_em_casa.pdf

[2] 2. Nishimura F, Carrara A, Freitas CE. Atendimento domiciliar e internações hospitalares: uma análise utilizando um desenho de regressão descontínua. In: Anais do 45. Encontro Nacional de Economia; 12-15 dez 2017; Natal, RN. Niterói, RJ: ANPEC; 2017 [citado 1 jan 2018]. Disponível em: https://www.anpec.org.br/encontro/2017/submissao/files_I/i12-484e2dab986f101b08458bba0820663e.pdf

[3] 3. Castro MSM, Travassos C, Carvalho MS. Efeito da oferta de serviços de saúde no uso de internações hospitalares no Brasil. Rev Saude Publica. 2005;39(2):277-84. 10.1590/S0034-89102005000200020 15895149

[4] 4. O’Dwyer GO, Oliveira SP, Seta MH. Avaliação dos serviços hospitalares de emergência do programa QualiSUS. Cienc Saude Coletiva. 2009;14(5):1881-90. 10.1590/S1413-81232009000500030 19851601

[5] 5. Bittencourt RJ, Hortale VA. Intervenções para solucionar a superlotação nos serviços de emergência hospitalar: uma revisão sistemática. Cad Saude Publica. 2009;25(7):1439-54. 10.1590/S0102-311X2009000700002 19578565

[6] 6. Farrero E, Escarravill J, Prats E, Maderal M, Manresa F. Impact of a hospital-based home-care program on the management of COPD patients receiving long-term oxygen therapy. Chest. 2001;119(2):364-9. 10.1378/chest.119.2.364 11171710

[7] 7. Amaral NN, Cunha MCB, Labronici RHDD, Oliveira ASB, Gabbai AA. Assistência domiciliar à saúde (Home Health Care): sua história e sua relevância para o sistema de saúde atual. Rev Neurocienc. 2001 [citado 1 jan 2018];9(3):111-7. Disponível em: http://revistaneurociencias.com.br/edicoes/2001/RN%2009%2003/Pages%20from%20RN%2009%2003-5.pdf

[8] 8. Silva KL, Sena RR, Feuerwerker LCM, Silva PM, Martins ACS. Desafios da atenção domiciliar sob a perspectiva da redução de custos/racionalização de gastos. Rev Enfermagem UFPE. 2014 [citado 1 jan 2018];8(6):1561-7. Disponível em: https://periodicos.ufpe.br/revistas/revistaenfermagem/article/view9846/10055

[9] 9. Sorensen AA, Mendes IAC, Hayashida M. Atendimento domiciliar: análise de um serviço privado. Rev RENE. 2004;5(2):86-92.

[10] 10. Gelman A, Imbens G. Why high-order polynomials should not be used in regression discontinuity designs. J Bus Econ Stat. 2019;37(3)447-56. 10.1080/07350015.2017.1366909

[11] 11. Angrist JD, Pischke JS. Mostly harmless econometrics: an empiricist’s companion. Princeton, NJ: Princeton University Press; 2008.

[12] 12. Thistlethwaite DL, Campbell DT. Regression discontinuity analysis: an alternative to the ex post facto experiment. J Educ Psychol. 1960;51(6):309-17. 10.1037/h0044319

[13] 13. Toro W, Tigre R, Sampaio B. Daylight Saving Time and incidence of myocardial infarction: evidence from a regression discontinuity design. Econ Lett. 2015;136:1-4. 10.1016/j.econlet.2015.08.005

[14] 14. Fujiwara T. Voting technology, political responsiveness, and infant health: evidence from Brazil. Econometrica. 2015;83(2):423-64. 10.3982/ECTA11520

[15] 15. Smith AC. Spring forward at your own risk: Daylight Saving Time and fatal vehicle crashes. Am Econ J Appl Econ. 2016;8(2):65-91. 10.1257/app.20140100

[16] 16. Calonico S, Cattaneo MD, Farrell MH, Titiunik R. Regression discontinuity designs using covariates. Rev Econ Stat. 2018 July. 10.1162/rest_a_00760

