# Supplementary material for: Effect of the Melhor em Casa program on hospital costs
Source: Rev Saude Publica. 2019 Nov 18;53:104. doi: 10.11606/s1518-8787.2019053000859 (PMC6863111; doi:10.11606/s1518-8787.2019053000859)
Supplement: Translation

# Translation: Efeito do programa Melhor em Casa sobre os gastos hospitalares

**Authors:** Fábio Nishimura, Aniela Fagundes Carrara, Carlos Eduardo de Freitas

**Affiliations:** I Universidade Federal de Mato Grosso. Curso de Ciências Econômicas. Programa de Pós-Graduação em Economia. Rondonópolis, MT, Brasil

**Keywords:** Serviços de Assistência Domiciliar, economia, Custos de Cuidados de Saúde, Avaliação de Programas e Projetos de Saúde, Sistema Único de Saúde

## Abstract

**OBJETIVO:**

Verificar se o programa Melhor em Casa consegue reduzir gastos com as internações hospitalares.

**MÉTODOS:**

Utilizamos como estratégia empírica um desenho de regressão descontínua, o que reduz problemas de endogeneidade de nosso modelo. Realizamos também testes de respostas heterogêneas e de robustez. Os dados da variável dependente, despesas ou gastos com internações hospitalares, foram coletados no Departamento de Informática do Sistema Único de Saúde (Datasus), usando o conjunto de microdados do Sistema de Internações Hospitalares do Sistema Único de Saúde referente ao período de 2010 a 2013, totalizando 3.609.384 observações nos três anos de análise. As covariáveis ou variáveis de controle utilizadas foram a idade e os gastos com pacientes na unidade de terapia intensiva, também coletadas no Datasus e referentes ao mesmo período.

**RESULTADOS:**

Os resultados apontam que o efeito do programa Melhor em Casa efetivamente reduziu os gastos com internações hospitalares em aproximadamente 4,7% em 2011, 5,8% em 2012 e 10,2% em 2013.

**CONCLUSÕES:**

A partir das análises, observamos que manter o programa é uma forma altamente eficaz de melhorar a gestão de recursos públicos, pois conseguiu reduzir as despesas com as internações nos três anos em análise. O programa obteve redução nas despesas com internação dos grupos de risco e também em situações que elevam as despesas hospitalares, como falta de equipamentos e internações eletivas. Assim, pode-se afirmar que o programa consegue reduzir as despesas com as internações, principalmente nos grupos com maior vulnerabilidade, mostrando-se eficiente como política pública.

## INTRODUÇÃO

O objetivo principal deste trabalho é verificar se o programa Melhor em Casa (daqui em diante, MemC) consegue reduzir os custos com internações hospitalares. Entende-se que o atendimento hospitalar é uma forma de política social na área da saúde de suma importância para a manutenção da vida e uma forma obrigatória de restabelecer os princípios constitucionais com a população brasileira. O atendimento hospitalar deve ser conduzido de forma suficientemente eficaz, para não ocasionar o colapso do sistema, principalmente em um momento de recursos orçamentários escassos.

Diante disso, para conter problemas referentes às internações hospitalares e reduzir os custos desnecessários para a população, o governo federal criou em 2011 o MemC^1 , 2^ . O programa visa uma articulação com a atenção domiciliar (AD) – ou seja, o paciente atendido na unidade de saúde e que está apto para concluir sua recuperação no conforto de seu lar, junto aos seus familiares, poderá ser transferido. Assim, há uma redução dos custos com a internação e diminuição da mortalidade dentro dos hospitais.

No entanto, atualmente não verificamos empenho dos gestores de saúde. O que se observa, de fato, são casos de má administração e más condições, tanto técnicas como estruturais, que reduzem a eficiência dos serviços hospitalares. As consequências são custos irrecuperáveis, tanto econômicos quanto ligados à vida^3^ .

Um dos grandes problemas do atendimento hospitalar são os custos dos serviços prestados na unidade de saúde, com níveis elevados de desperdício, o qual chega à cifra de R$ 3,6 bilhões por ano gastos com consultas, internações e exames desnecessários. A composição complexa dos custos hospitalares propicia o desperdício e gera ineficiência no âmbito do serviço público^9^ . Dessa forma, a contribuição deste trabalho, além de verificar o efeito do MemC, também está na aplicação da estratégia empírica do modelo de regressão descontínua (RD), que garante respostas sem viés endógeno dos estimadores, confirmando ou não a eficiência do programa.

Os testes de repostas heterogêneas e de robustez foram empregados para verificar se os estimadores foram precisos. A hipótese principal do estudo era a de que o programa consegue reduzir os custos com as internações hospitalares, tornando a gestão hospitalar pública mais eficiente.

## MÉTODOS

Os dados da variável dependente, despesas ou gastos com internações hospitalares, foram coletados no Departamento de Informática do Sistema Único de Saúde (Datasus), usando o conjunto de microdados do Sistema de Internações Hospitalares do Sistema Único de Saúde (SIH-SUS) referente ao período de 2010 a 2013, totalizando 3.609.384 observações nos três anos de análise. Os valores da variável de interesse, despesas por internação hospitalar, foram deflacionados; assim, a série sofre impacto real, sem influência inflacionária.

No ano de 2011, nos municípios onde existe o programa, houve um gasto médio com internação de R$ 38,67 por paciente/dia. Em 2013, esse valor passou a ser de R$ 42,78 por paciente/dia, um aumento de 10,6% em três anos. Nos municípios que não recebem o programa, a média de gastos era de R$ 30,83 por paciente/dia em 2011 e de R$ 33,51 por paciente/dia em 2013, com um aumento de 8,7% em três anos. Esses dados apontam que existe uma motivação para que o programa seja implantado onde o gasto com internação é maior.

Para analisar os efeitos do MemC, foram coletados dados do Datasus e do Cadastro Nacional de Estabelecimentos de Saúde (CNES), ambos órgãos do Ministério da Saúde. Como forma de identificar os municípios que recebem o MemC, utilizou-se uma *dummy* em que eles foram denominados “tratados” e receberam o valor 1, enquanto os municípios que não receberam o MemC foram denominados “controles” e recebem o valor 0.

Pelo conjunto de dados obtidos, observamos que em 2011 o programa contemplou 23 dos 5.570 municípios brasileiros existentes. Já em 2012 esse número saltou para 90 municípios, e em 2013 a quantidade de municípios contemplados passou a ser de 184, evidenciando um salto de 0,41% de cobertura do programa em 2011 para 3,3% em 2013.

As covariáveis ou variáveis de controle utilizadas foram a idade e os gastos com pacientes na unidade de terapia intensiva. Esses dados foram coletados no banco de dados Datasus, referentes ao período de 2010 a 2013. É importante frisar que as variáveis de controle são utilizadas para dar maior precisão aos valores estimados^10^ , mas não devem interferir ou gerar qualquer viés nos resultados encontrados do modelo.

Neste trabalho, buscamos compreender o efeito da atenção domiciliar (neste caso, do programa MemC) sobre o valor das despesas com as internações hospitalares. Como estratégia empírica, utilizamos o desenho de regressão descontínua^11^ pois o programa possui um critério de adesão relacionado a um ponto de corte e de probabilidade exógeno, que chamamos de *cutoff* . O *cutoff* estabelecido foram municípios com mais de 20.000 habitantes. Assim, estimamos a seguinte equação:

$$Y_{imp}\;=\;\beta_{0}\;+\;\beta_{1}\;MemC_{imp}\;+\;\beta_{2}\;T_{imp}\;+\;\varepsilon_{imp}$$ (1)

onde *Y*_*imp*_ é a variável de interesse do modelo para o indivíduo *i* , no município *m* e no ano *p* ; *MemC* é o programa Melhor em Casa, que leva valor igual a 1, caso o município do indivíduo receba o programa, e 0 caso contrário, para o indivíduo *i,* no município *m* e no ano *p* ; o *T*_*ip*_ indica se o município está acima ou abaixo do *cutoff* , citado anteriormente, para o indivíduo *i,* no município *m* e no ano; por fim, ε_*ip*_ é um termo de erro. Devido à sensibilidade de considerar um aumento de probabilidade – mas não de zero para um, pois a atribuição ao tratamento pode depender de fatores adicionais –, aplica-se o modelo de regressão descontínua *fuzzy* . Isso garante uma identificação causal entre as variáveis selecionadas, possibilitando a confirmação do efeito do MemC sobre as despesas hospitalares com maior segurança estatística.

Para realizar as regressões locais, é preciso introduzir uma janela ( *bandwidth* ) que determinará o tamanho da distância do ponto de corte no nível de observações da amostra. Utilizamos, então, a metodologia de Calonico et al.^16^ Essa variação de *bandwidth* também é uma forma de garantir nossa estratégia empírica, ressaltando que testamos o nosso modelo para uma especificação linear e quadrática.

Além dos testes já descritos, aplicamos mais três testes de robustez para confirmar nossa estratégia empírica. Primeiramente verificamos possíveis efeitos oriundos de outros programas ou ações de anos anteriores ao início do programa. Outro teste foi a alteração de *cutoff,* ou seja, fizemos nossas regressões com *cutoffs* diferentes do estipulado pelo programa. Finalmente, testamos as covariáveis de nosso modelo. Os testes não devem gerar estimadores estatisticamente significantes, garantindo os parâmetros de nosso modelo.

Elaboramos também testes para respostas heterogêneas, buscando a compreensão e confirmação dos efeitos do MemC em situações de subamostras, com o que denominamos grupos de risco (grávidas e idosos), que necessitam de um tempo maior de internação. Procuramos analisar se o programa consegue afetar os municípios com um número efetivo de equipamentos menor que a média nacional, o que provocaria um maior tempo de internação devido à demora dos resultados dos exames. Por último, verificamos o efeito do MemC para o grupo de pessoas internadas sem caráter de urgência, ditas eletivas, o que demonstraria a rapidez no cuidado e a redução dos custos com esse tipo de hospitalização.

## RESULTADOS

Para garantir os resultados estatísticos, inicialmente verificamos se ocorre descontinuidade no ponto de corte de 20.000 habitantes. O resultado desse teste inicial é apresentado na Figura , na qual observamos que existe uma descontinuidade: uma redução no valor gasto com internações hospitalares. Ela é de suma importância, pois esse critério garante que nossos resultados advêm de uma relação causal e da comparação que podemos realizar entre municípios que estão próximos ao ponto de corte (o ponto 0 da Figura ) e que têm como diferença apenas a existência ou não do programa.

**Figura:** Descontinuidade do programa no *cutoff* .

A existência da descontinuidade, fator básico para que possamos calcular nossos estimadores, é confirmada na Tabela 1 , na qual os resultados de todas as regressões realizadas apontam estimadores negativos e significantes estatisticamente. As reduções nos valores das despesas com internações hospitalares chegaram a 4,7% (p < 0,01). Esse efeito se manteve nos anos subsequentes, atingindo uma redução de até 10,2% (p < 0,01).

**Tabela 1:** Efeito do programa Melhor em Casa sobre os gastos com internações hospitalares 2010 a 2013.

Variável	1	2	3	4	5	6
*MemC*_t_	-0,044^h^	-0,047^h^	-0,007	-0,035^j^	-0,034^j^	-0,007
	(0,016)	(0,016)	(0,005)	(0,020)	(0,020)	(0,007)
*MemC*_t+1_	-0,048^i^	-0,058^h^	-	-0,044^i^	-0,055^i^	-
	(0,019)	(0,021)		(0,023)	(0,024)	
*MemC*_t+2_	-0,101^h^	-0,102^h^	-	-0,098^h^	-0,096^h^	-
	(0,018)	(0,019)		(0,019)	(0,020)	
						
Especificação	Linear	Linear	Linear	Quad	Quad	Quad
*Bandwidth*	msetwo	msecomb2	msetwo	msetwo	msecomb2	msetwo
Controles	Sim	Sim	Sim	Sim	Sim	Sim

N. Obs	182.350	182.350	182.350	182.350	182.350	182.350

^a^Variável dependente: log do valor de internação.

^b^Todas as especificações usam Kernel triangular.

^c^O MemC (Melhor em Casa) estima a descontinuidade de municípios logo acima de 20.000 habitantes.

^d^Msetwo: *two different MSE-optima l bandwidth selectors* e Msecomb2: *two different MSE-optima l bandwidth selectors* for median, onde referem-se a seletores de *bandwidth* ótimo de Calonico et al.^16^

^e^As colunas 1, 2, 4 e 5 representam as estimações principais de nosso modelo especificado. As colunas 3 e 6 referem-se a um teste de falsificação de um ano antes do início do MemC.

^f^Erro-padrão robusto em parênteses.

^g^Nível de significância estatística representado por: (h) p < 0,01, (i) p < 0,05 e (j) p < 0,10.

^l^Quad: função quadrática

Em relação às respostas heterogêneas, pelos estimadores apresentados no Painel A, da Tabela 2 , conclui-se que o programa reduziu os valores gastos com internações de mulheres com gravidez de risco, no período de três anos após seu início, resultando em uma economia de até 9,3% (p < 0,01). No painel B, relativo ao grupo de pessoas idosas, também houve uma redução de até 9,6% (p < 0,01) dentro do período de três anos. O programa mostra, dessa forma, que a redução nos gastos atinge grupos de risco e com maior vulnerabilidade.

**Tabela 2:** Efeito do programa Melhor em Casa sobre os gastos com internações hospitalares – mulheres com gravidez de risco e idosos, 2011 a 2013.

Variável	1	2	3	4
**Painel A:** Efeito sobre gestantes com risco				
*MemC*_t_	-0,037^h^	-0,040^g^	-0,041^g^	-0,042^g^
	(0,016)	(0,016)	(0,017)	(0,017)
*MemC*_t+1_	-0,048^h^	-0,059^g^	-0,045^h^	-0,055^h^
	(0,021)	(0,021)	(0,023)	(0,024)
*MemC*_t+2_	-0,092^g^	-0,090^g^	-0,093^g^	-0,092^g^
	(0,018)	(0,019)	(0,019)	(0,020)

N. Obs.	166.295	166.295	166.295	166.295

**Painel B:** Efeito sobre idosos				
*MemC*_t_	-0,067^h^	-0,067^g^	-0,073^h^	-0,074^h^
	(0,031)	(0,015)	(0,037)	(0,037)
*MemC*_t+1_	-0,073^h^	-0,064^i^	-0,085^g^	-0,062^i^
	(0,033)	(0,036)	(0,034)	(0,036)
*MemC*_t+2_	-0,096^g^	-0,111^g^	-0,086^h^	-0,079^i^
	(0,035)	(0,040)	(0,040)	(0,043)
Especificação	Linear	Linear	Quad	Quad
*Bandwidth*	msetwo	msecomb2	msetwo	msecomb2
Controles	Sim	Sim	Sim	Sim

N. Obs.	166.295	166.295	166.295	166.295

^a^Todas as especificações usam Kernel triangular.

^b^O MemC (Melhor em Casa) estima a descontinuidade de municípios logo acima de 20.000 habitantes.

^c^Msetwo: *two different MSE-optima l bandwidth selectors* e Msecomb2: *two different MSE-optima l bandwidth selectors* for median, onde referem-se a seletores de *bandwidth* ótimo de Calonico et al.^16^

^d^As colunas 1,2, 3 e 4 representam estimações como respostas heterogêneas.

^e^Erro-padrão robusto em parênteses.

^f^Nível de significância estatística representado por: (g): p < 0,01, (h): p < 0,05 e (i): p < 0,10.

^j^Quad: função quadrática

No Painel A da Tabela 3 , verifica-se que nos primeiros três anos a redução dos valores das despesas com internações hospitalares oscilou entre 4,4% e 11,7%. Ambas reduções foram significantes estatisticamente a 1%, mesmo considerando o município com quantidade de equipamentos abaixo da média nacional – o que dificulta a liberação do paciente, pois também atrasa a entrega dos resultados dos exames, gerando maiores despesas. No Painel B da Tabela 3 , há uma redução entre 6,7% (p < 0,1) e 17,9% (p < 0,01), indicando uma economia nas despesas com internações eletivas.

**Tabela 3:** Efeito do programa Melhor em Casa sobre os gastos com internações hospitalares – municípios com menos equipamentos hospitalares e internações eletivas, 2011 a 2013.

Variável	1	2	3	4
**Painel A:** Efeito sobre municípios com menos equipamentos hospitalares				
*MemC*_t_	-0,049^g^	-0,055^g^	-0,046^h^	0,044^h^
	(0,016)	(0,016)	(0,020)	(0,020)
*MemC*_t+1_	-0,048^h^	-0,057^g^	-0,044^i^	-0,055^h^
	(0,021)	(0,021)	(0,023)	(0,024)
*MemC*_t+2_	-0,110^g^	0,117^g^	-0,106^g^	-0,101^g^
	(0,018)	(0,020)	(0,019)	(0,019)

N. Obs.	182.350	182.350	182.350	182.350

**Painel B:** Efeito sobre internações eletivas				
*MemC*_t_	-0,085^g^	-0,071^h^	-0,161^g^	-0,134^g^
	(0,032)	(0,030)	(0,041)	(0,041)
*MemC*_t+1_	-0,067^i^	-0,068^i^	-0,075^i^	-0,085^i^
	(0,038)	(0,038)	(0,040)	(0,044)
*MemC*_t+2_	-0,125^g^	-0,122^g^	-0,171^g^	-0,179^g^
	(0,042)	(0,046)	(0,047)	(0,047)
Especificação	Linear	Linear	Quad	Quad
*Bandwidth*	msetwo	msecomb2	msetwo	msecomb2
Controles	Sim	Sim	Sim	Sim

N. Obs.	46.916	46.916	46.916	46.916

^a^Todas as especificações usam Kernel triangular.

^b^O MemC (Melhor em Casa) estima a descontinuidade de municípios logo acima de 20.000 habitantes.

^c^Msetwo: *two different MSE-optima l bandwidth selectors* e Msecomb2: *two different MSE-optima l bandwidth selectors* for median, onde referem-se a seletores de *bandwidth* ótimo de Calonico et al.^16^

^d^As colunas 1, 2, 3 e 4 representam estimações como respostas heterogêneas.

^e^Erro-padrão robusto em parênteses.

^f^Nível de significância estatística representado por: (g): p < 0,01, (h): p < 0,05 e (i): p < 0,10.

^j^Quad: função quadrática

Para garantir estatisticamente a afirmação da hipótese inicial, além das respostas heterogêneas, aplicamos testes de robustez. Na Tabela 1 , colunas 3 e 6, fizemos o teste de possível ocorrência de efeitos em anos anteriores ao início do programa. Como resultado, observa-se que não há significância estatística, não se verificando indícios de que ocorra algum efeito por parte de qualquer fator externo e anterior ao início do MemC nas despesas com internações. Reforça-se assim a ideia de que o programa é o principal fator para a redução das despesas.

Na Tabela 4 , continuamos com os testes de robustez. Nos painéis A e B, verifica-se que, ao criar falsos *cutoffs,* de 10.000 habitantes e de 50.000 habitantes, os resultados estimados não têm significância estatística. Isso era esperado, pois certifica que os efeitos acontecem apenas no critério de corte determinado pelo programa, que é de 20.000 habitantes, demonstrando que não se trata de acaso estatístico.

**Tabela 4:** Testes de robustez do modelo especificado, 2011 a 2013.

Variável	1	2	3	4
**Painel A:** Alteração do *cutoff* para 10.000 habitantes				
*MemC*_t_	-0,020	-0,029	-0,005	-0,031
	(0,015)	(0,020)	(0,017)	(0,021)
*MemC*_t+1_	0,009	-0,016	0,033	-0,006
	(0,041)	(0,055)	(0,041)	(0,068)
*MemC*_t+2_	0,026	0,052	0,046	-0,048
	(0,035)	(0,037)	(0,039)	(0,053)

N. Obs.	215.393	215.393	215.393	215.393

**Painel B:** Alteração do *cutoff* para 50.000 habitantes				
*MemC*_t_	0,012	0,002	0,033	0,028
	(0,020)	(0,017)	(0,021)	(0,019)
*MemC*_t+1_	-0,0002	-0,018	0,038	0,005
	(0,033)	(0,061)	(0,035)	(0,094)
*MemC*_t+2_	0,029	0,025	0,041	0,033
	(0,022)	(0,022)	(0,026)	(0,024)

N. Obs.	400.834	400.834	400.834	400.834

**Painel C:** Teste sobre as covariáveis				
Idade	-0,013	0,357	-0,051	3,482
	(1,842)	(1,698)	(2,423)	(2,460)
Dias de internação na UTI	0,090	0,092	0,096	0,102
	(0,056)	(0,054)	(0,075)	(0,072)
Especificação	Linear	Linear	Quad	Quad
*Bandwidth*	msetwo	msecomb2	msetwo	msecomb2
Controles	Sim	Sim	Sim	Sim

N. Obs.	182.350	182.350	182.350	182.350

^a^Todas as especificações usam Kernel triangular.

^b^O MemC (Melhor em Casa) estima a descontinuidade de municípios com mais de 20.000 habitantes, para os casos do Painel C.

^c^Msetwo: *two different MSE-optima l bandwidth selectors* e Msecomb2: *two different MSE-optima l bandwidth selectors* for median, onde referem-se a seletores de *bandwidth* ótimo de Calonico, Cattaneo e Farrel^16^

^d^As colunas 1, 2, 3 e 4 representam estimativas como testes de robustez.

^e^Erro-padrão robusto em parênteses.

^f^Nível de significância estatística representado por: (g): p < 0,01, (h): p < 0,05 e (i): p < 0,10.

^j^Quad: função quadrática

Finalizando nossos testes, no Painel C da Tabela 4 , testamos as variáveis de controle do modelo, mas não obtivemos significância estatística em nenhum estimador, o que estabelece que o único fator a promover efeitos na redução dos valores com as despesas de internações advém do MemC. Assim, após verificar todos os resultados estimados, podemos concluir que o programa gera uma redução dos valores com despesas relativas às internações hospitalares.

## DISCUSSÃO

O presente estudo analisou os efeitos do Melhor em Casa sobre os gastos com internações hospitalares. O programa propõe a prestação de serviços, por parte de equipes compostas de diversos profissionais da área de saúde, para promover a recuperação do enfermo no conforto de seu lar ( *home care* ). Além da recuperação em um ambiente mais saudável para o paciente, também promove a desocupação de leitos, e com isso, consegue uma redução das despesas diárias com a internação.

Após cuidados metodológicos que garantem que os resultados estimados do trabalho apresentem características causais, verificamos que o MemC consegue ser efetivo em relação à diminuição das despesas com internações hospitalares, com reduções de aproximadamente 4,7% em 2011, 5,8% em 2012 e 10,2% em 2013. Para dar sustentação estatística a nossos estimadores, verificou-se que todos os resultados, tanto das respostas heterogêneas quanto dos testes de robustez, foram os esperados, confirmando os resultados de nosso modelo.

Podemos observar que os grupos de risco (mulheres grávidas e idosos), mesmo com gastos maiores devido aos cuidados específicos, também têm gastos reduzidos pelo programa. Quanto aos municípios com um número de equipamentos hospitalares menor do que a média nacional, verificou-se que o MemC consegue ser efetivo e também reduz as despesas com as internações. Nos casos relativos às internações eletivas, ou seja, aquelas que não são de urgência, o programa também consegue ser eficiente. Dessa forma, pode-se afirmar que o programa ajuda na redução das despesas com as internações, principalmente em grupos de risco e com maior vulnerabilidade, mostrando-se uma política pública eficiente.
